# A systematic review and meta-analysis on tubal ligation and breast cancer risk

**DOI:** 10.1186/s13643-022-02000-8

**Published:** 2022-06-19

**Authors:** Nazila Najdi, Arezoo Esmailzadeh, Maryam Shokrpour, Somayeh Nikfar, Seyedeh Zahra Razavi, Mahdi Sepidarkish, Saman Maroufizadeh, Saeid Safiri, Amir Almasi-Hashiani

**Affiliations:** 1 Department of Obstetrics and Gynecology, Arak University of Medical Sciences, Arak, Iran; 2 Department of Obstetrics and Gynecology, Trauma Research Center, Baqiyatallah University of Medical Sciences, Tehran, Iran; 3 Zanjan University of Medical Sciences, Zanjan, Iran; 4 Department of Biostatistics and Epidemiology, Babol University of Medical Sciences, Babol, Iran; 5 Department of Biostatistics, School of Health, Guilan University of Medical Sciences, Rasht, Iran; 6 Research Center for Integrative Medicine in Aging, Aging Research Institute, Tabriz University of Medical Sciences, Tabriz, Iran; 7 Social Determinants of Health Research Center, Department of Community Medicine, Faculty of Medicine, Tabriz University of Medical Sciences, Tabriz, Iran; 8 Department of Epidemiology, School of Health, Arak University of Medical Sciences, Golestan St., Arak, Iran; 9 Traditional and Complementary Medicine Research Center, Arak University of Medical Sciences, Golestan St., Arak, Iran

**Keywords:** Breast neoplasm, Breast cancer, Tubal sterilization, Tubal ligation, Meta-analysis

## Abstract

**Background:**

Based on previous studies, it has been hypothesized that tube sterilization may be associated with a lower risk of breast cancer. This study aims to investigate the relationship between tubal ligation and the risk of breast cancer through a systematic review and meta-analysis.

**Methods:**

In this systematic review and meta-analysis, PubMed/Medline, Web of Science, Scopus, and Google Scholar were searched for relevant non randomized studies published up to November 2020. Then, we screened the papers to include the eligible papers in the meta-analysis. Finally, we pooled the extracted results of individual studies to estimate the summary effect size. All analyses were done using Stata software version 13 (Stata Corp, College Station, TX).

**Results:**

Four hundred sixty-four papers were retrieved from PubMed/Medline (160), Scopus (165), and Web of Science (139), and 21 papers from Google Scholar and manual search of references in selected full texts. After the removal of duplicates and screening of the papers, 11 articles (6 cohort and 5 case-control study) were included in the final analysis. The results of cohort (RR = 0.99, 95% CI = 0.97–1.0, *I*^2^ = 21.1%) and case control studies (OR = 0.87, 95% CI = 0.62–1.12, *I*^2^ = 88.9%) revealed that tubal ligation was not significantly associated with breast cancer risk.

**Conclusion:**

According to our findings, tubal ligation cannot be considered as a risk factor associated with breast cancer risk.

**Supplementary Information:**

The online version contains supplementary material available at 10.1186/s13643-022-02000-8.

## Background

Permanent contraception, also known as female sterilization or tubal ligation, is a surgical procedure in which fallopian tubes are blocked or removed. This contraceptive method is one of the most popular methods of family planning around the world [1, 2], accounting for 21.8% of contraceptive users in 2014 after oral contraceptives by 25.3% [3]. The prevalence of tubal ligation use has increased to 24% in 2019, with 219 million women worldwide choosing this method as their favorite contraceptive [3, 4].

Based on previous studies, it has been hypothesized that tube sterilization may be associated with a lower risk of breast cancer [2], especially among women < 45 years of age [5]. Ovarian hormones are involved in the pathogenesis of breast cancer. Therefore, tubal ligation may alter the risk of breast cancer due to changes in hormone levels before menopause age [5]. However, tubal ligation can also damage surrounding tissues, disrupting blood flow to ovaries and their hormonal function, thereby reducing the risk of breast cancer [6].

Numerous case-control and cohort studies [5–9] have been conducted in different parts of the world with different methodologies to examine this relationship. The findings, however, have been inconsistent and there is still no consensus on the effect of tubal ligation on breast cancer risk. Although most studies have reported either no association or reduced risk of breast cancer following tubal ligation, there is also some evidence of an increased risk of breast cancer [9]. Besides, a study has reported that there is an inverse relationship between tubal ligation and breast cancer mortality [7].

In previous meta-analysis studies, the protective effect of tubal ligation on endometrial [10] and ovarian cancers [11, 12] was reported. Therefore, it is expected that tubal ligation, with a similar mechanism, can be associated with a reduction in breast cancer risk.

With the inconsistency of previous study findings and also the fact that the last meta-analysis on this topic was conducted a decade ago when due to lack of evidence only PubMed database was searched [2], this study aimed to investigate the relationship between tubal ligation and risk of breast cancer through a systematic review and meta-analysis on non-randomized studies.

## Methods

### Study design

The systematic review and meta-analysis were conducted following the standard guideline of “Preferred Reporting Items for Systematic Reviews and Meta-Analyses (PRISMA)” [13] and “Cochrane Handbook for Systematic Reviews of Interventions” [14].

### Search strategy

To extract the relevant articles, a specific search strategy was devised, using a variety of keywords, for the following three international databases: Medline via PubMed, Scopus, and Web of Science. Medical Subject Heading (MeSH) and our keywords (including “breast neoplasms,” “breast cancer,” “sterilization, tubal,” “tubal sterilization,” “tubal ligation,” “tubal occlusion,” “female sterilisation,” “sterilization, reproductive,” and “reproductive sterilization”) were searched in PubMed. The search was filtered to only subsume human and English language studies. The modified keywords were then used to search in Scopus and Web of Science databases. More detail on the search strategy is reported in Supplementary file 1. The searching activities were conducted by authors (AAH, SS, and SZR) on November 23. Finally, Google Scholar was searched for gray literature, and references of selected articles were also manually checked by authors to find the relevant articles.

### Study selection

To select the relevant articles, the retrieved articles were entered into Endnote version X8. Duplicate articles were removed in this stage. Then, the titles and abstracts of the remaining articles were screened and the irrelevant articles were excluded. After that, the full texts of the remaining articles were evaluated and unrelated articles were discarded. The required information was finally extracted from the remaining related articles. In cases where an article was relevant but the necessary data was not reported, the corresponding author was contacted. All stages of the study selection were performed by two people (AAH and SM). In the case of controversies, decisions were made in consultation with other authors.

### Inclusion and exclusion criteria

Non-randomized articles that were published in the English language, up to November 2020, and examining the association between tubal ligation and breast cancer were included in the study. Moreover, only case-control and cohort studies were included, and other types of articles namely randomized interventional studies, letters to the editors, case reports, case series, editorials, review articles, and commentaries were excluded.

### Data extraction

Data were extracted from the full-text of retrieved articles by the authors (AAH, MS2, SM, and SZR), and in the cases of disagreements, decisions were made in consultation with other authors. Data extracted from each article included first author’s name, year of publication, study design, type of effect size (e.g., odds ratio (OR) and risk ratio (RR)), effect size (and its 95% confidence interval (95% CI)), place of study, the quality score of the article, and studied population. In cohort studies, RR was extracted, and in case-control studies, OR was extracted.

### Risk of bias

Qualitative evaluation of the included studies was performed by two authors based on the Newcastle-Ottawa Quality Assessment Scale (NOS), adapted for case-control and cohort studies. This scale is introduced to assess the quality of observational studies. The NOS evaluates each study based on six items in three overarching headings of selection, comparability, and exposure. Each item is given a star and its score ranges from 0 to 9. The articles were finally categorized into three groups with a score of more than 6 as high, 3 to 6 as moderate, and less than 3 as low quality.

### Statistical analysis

To check the heterogeneity across the studies, the *I*^2^ statistic with a chi-square test was used. Also, in cases where there was significant heterogeneity, the random-effect model was used, and in other cases, the fixed-effect model was conducted to pool the effect sizes with “metan” command [15]. To explore the publication bias, Egger’s linear regression, Begg’s test (with “metabias” command [16]), and funnel plot (with “metafunnel” command [17]) were used. Moreover, a sensitivity analysis was performed to identify the effect of each study on the summary effect size with “metainf” command [18]. All analyses were done using Stata software version 13 (Stata Corp, College Station, TX, USA).

## Results

### Study selection and study characteristics

**Fig. 1:**
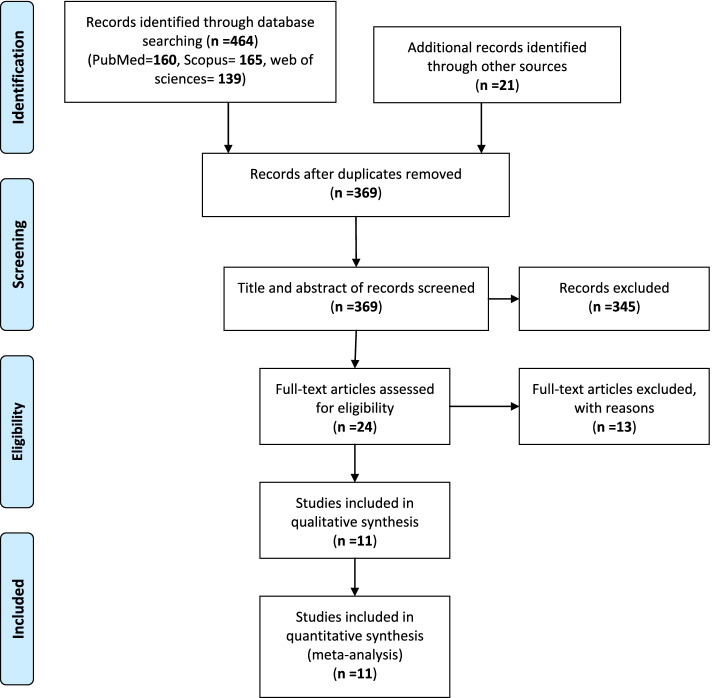
Flow diagram of the literature search for studies included in the meta-analysis

The PRISMA flow diagram regarding the selection of the articles is displayed in Fig. 1. Searching the three international databases, we retrieved 464 papers (PubMed/Medline: 160, Scopus: 165, and Web of Science: 139). Twenty-one papers were retrieved from Google Scholar and a manual search of the references of all selected full texts. After the removal of duplicate papers, 369 papers remained from which 345 papers were excluded following their titles and abstracts screening. Then, the full texts of the remaining 24 articles were evaluated, and 13 articles were excluded due to irrelevance, lack of meeting inclusion criteria, or lack of sufficient data reporting. Finally, 11 articles [2, 5, 6, 8, 9, 19–24] were included in the meta-analysis.

The eleven studies that met the inclusion criteria are summarized in Table 1. Five were case-control [5, 9, 19–21] and six were cohort study designs [2, 6, 8, 22–24]. The oldest study was published in 1988 [9] and the latest in 2016 [24]. There were 6 articles from the USA [2, 5, 6, 8, 9, 21], 3 articles from Europe [19, 22, 24], and 2 articles from Asia [20, 23]. The adjusted effect size was reported in all the articles except for a study from Korea. More details about the selected studies are reported in Table 1.

**Table 1:** Characteristics of the primary studies included in the meta-analysis

First author	Year	Study design	Country	Population	Quality Score*	TES	ES	LL	UL	Adjusted for:
Brinton L et al.	2000	Case Control	USA	Women 20–44 years	High	OR	1.09	0.9	1.3	Race, age at first birth, number of births, and years of education
Braga C et al.	1996	Case Control	Italy	Women 23–74 years	High	OR	0.6	0.3	1.3	Age, center, education, parity, age at first birth, menopausal status/age at menopause, age at menarche, history of benign breast disease, family history of breast cancer, and use of oral contraceptives
Press DJ et al.	2011	Case Control	USA	Women 35–64 years	High	OR	0.93	0.84	1.03	Age, race, study site, age at menarche, first-degree family history of breast cancer, number of term pregnancies, educational status, and duration of hormone therapy use
Dorjgochoo T et al.	2009	Cohort	China	Women 40–70 years	High	RR	1.22	0.95	1.55	
Eliassen AH et al.	2006	Cohort	USA	Women 30–55 years	High	RR	0.95	0.88	1.03	Multivariate-adjusted relative risk for time in 2-year periods, age in months, age at menarche, parity, age at first birth, height, weight change since age 18, body mass index at age 18, first-degree family history of breast cancer, benign breast disease, alcohol consumption, physical activity, oral contraceptive use, menopausal status and type of menopause, and use of postmenopausal hormones
Gaitskell K et al.	2016	Cohort	UK	Women 50–64 years	High	RR	0.99	0.97	1.01	Age, region, socioeconomic status, parity, age at first birth, hysterectomy, smoking, alcohol intake, physical activity, body mass index, and use of the oral contraceptive pill or menopausal hormones
Gaudet MM et al.	2013	Cohort	USA	Women 50–74 years	High	RR	1.06	0.69	1.18	
Shin MH et al.	1996	Case Control	Korea			OR	0.37	0.19	0.68	
Irwin KL et al.	1988	Case Control	USA	Women 20–54 years	High	OR	1.2	1.0	1.3	Age, data collection center, parity, menopausal status, family history of breast cancer, and history of benign breast disease
Iversen L et al.	2007	Cohort	UK	Women who were using oral contraceptives	High	RR	1.21	0.70	2.10	
Nichols H et al.	2013	Cohort	USA	Women 35–74 years	High	RR	0.91	0.82	1.02	

*TES* type of reported effect size, *ES* effect size, *UL* upper limit, *LL* lower limit, *OR* odds ratio, *RR* risk ratio

*Based on the Newcastle-Ottawa scale

### Risk of bias within studies

The quality of each study was checked according to the NOS and all studies were of high quality. However, we could not check the quality of a study from Korea [20] due to a lack of access to the full-text.

### Quantitative data synthesis

**Fig. 2:**
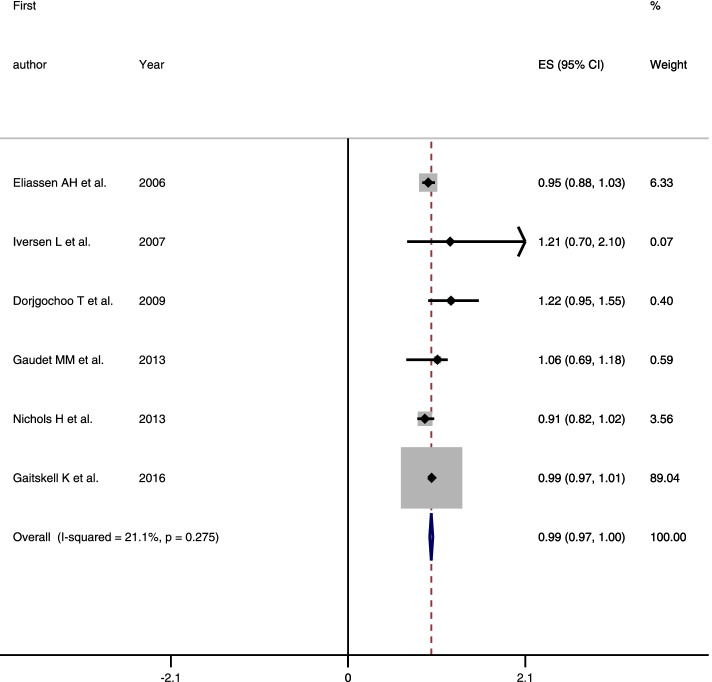
Forest plot describing the association between tubal ligation and breast cancer risk among cohort studies

**Fig. 3:**
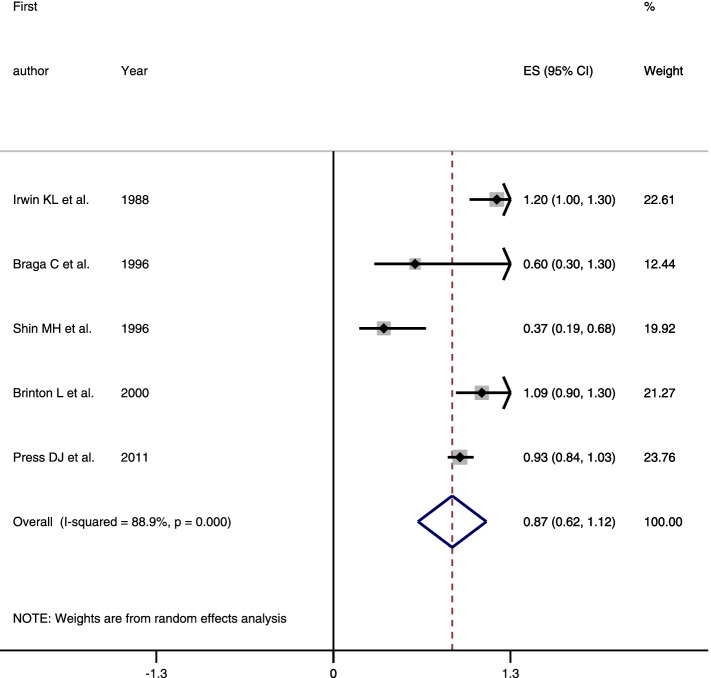
Forest plot describing the association between tubal ligation and breast cancer risk among case-control studies

In this study, the results of cohort and case-control studies was not combined together and the results pooled separately. Because of significant heterogeneity between primarily included case-control studies, a random-effects model with a Mantel-Hansel approach was used to summarize the findings, while cohort studies were combined using the fixed-effects model. The results of cohort (RR = 0.99, 95% CI = 0.97–1.0, *I*^2^ = 21.1%) and case control studies (OR = 0.87, 95% CI = 0.62–1.12, *I*^2^ = 88.9%) revealed that tubal ligation was not significantly associated with breast cancer risk (Figs. 2 and 3).

### Heterogeneity and meta-regression

The results suggested a significant heterogeneity between primary case control studies (heterogeneity chi-squared = 36.1, (d.f. = 4), *p* = 0.001, *I*^2^ (variation in ES attributable to heterogeneity) = 88.9%, estimate of between-study variance tau-squared = 0.066), and the results revealed that there is no significant heterogeneity among cohort studies (heterogeneity chi-squared = 6.3, (d.f. = 5), *p* = 0.275, *I*^2^ (variation in ES attributable to heterogeneity) = 21.1%, estimate of between-study variance tau-squared = 0.001). Therefore, a random-effects model was used to summarize the effect sizes in case control studies and fixed-effects model was used in cohort studies.

### Additional analysis

Based on previous evidence [25–27], the OR is a good estimate of the RR if the incidence of the disease is rare. Assuming that breast cancer is rare in people with and without a history of tubal ligation, we combined the effect sizes of all studies (11 studies). Overall, the pooled effect size in the meta-analysis revealed that tubal ligation was not significantly associated with breast cancer risk (summary effect size (SES) = 0.97, 95% CI = 0.89–1.04, *I*^2^ = 76.8%, random-effects model).

### Risk of bias across studies

Begg’s and Egger’s tests were used to check the possibility of publication bias. The findings, however, suggested that there was no evidence of publication bias among case-control (Begg’s test *p* value = 0.462, Egger’s test *p* value = 0.36) and cohort studies (Begg’s test *p* value = 0.133, Egger’s test *p* value = 0.34).

### Sensitivity analysis

Based on sensitivity analysis, the results showed that in both cohort and case-control studies, there was no association between tubal ligation and breast cancer risk by removing each individual studies. Also, overall sensitivity analysis showed that the highest estimate of summary effect size was related to the time when Shin et al. study [20] was excluded from the meta-analysis (SES = 1.01, 95% CI = 0.95–1.07). The lowest estimate on the other hand was related to the time when Irwin et al. study [9] was excluded from the meta-analysis (SES = 0.96, 95% CI = 0.89–1.02).

## Discussion

In this study, we collected and analyzed non-randomized articles examining the relationship between tubal ligation and breast cancer risk in a systematic review and meta-analysis. The results of the study strongly support the lack of an association between tubal ligation and breast cancer risk. Regardless of the statistically significant level, the estimated summary effect size was close to its null value of one, suggesting that the observed association was not clinically important (i.e., not in favor of a protective effect of tubal ligation on breast cancer risk). In addition, the small confidence intervals obtained from the models implicate accurate results.

Given that tubal ligation has a significant effect on reducing the risk of ovarian [11, 12] and endometrial cancers [10], our hypothesis in this study was that this association, with a similar mechanism, might be established for breast cancer too. On the contrary, we showed that there was no sufficient evidence to support this hypothesis, and it was concluded that tubal ligation is not associated with breast cancer risk. The lack of association between tubal ligation and breast cancer risk reinforces a hypothesis that the association between tubal ligation and ovarian cancer might be due to a mechanical barrier against ascending carcinogenic agents or due to screening effect (i.e., selective removal of suspicious ovaries during tubal ligation) [2].

From a total of 11 articles included in the meta-analysis, one study showed a significant increase [9] and one a significant decrease [20] in breast cancer following tubal ligation. The rest of the studies reported that there was no such association according to their findings. In fact, in line with our study, several studies have reported that tubal ligation cannot reduce the risk of breast cancer. In one study conducted by Calle et al. [7], however, the relationship between tubal ligation and the mortality rate of breast cancer was examined, and the authors concluded that there was an inverse relationship between tubal ligation and breast cancer mortality rate. To be precise, they reported that breast cancer mortality rate in those with a history of tubal ligation was 0.82 times higher than that of the control group, especially among women who were sterilized before age 35.

Previous studies have also suggested that women who have a history of tubal ligation are more prone to uterine surgery and hysterectomy [28], and bilateral oophorectomy [29], and these issues can act as a factor to reduce the risk of breast cancer. Given this, it can be hypothesized that tubal ligation alone may not play a role in reducing the risk of breast cancer but rather the surgery that occurs after tubal ligation might be a factor.

One of the important issues in meta-analysis studies is that the unadjusted data of individual studies are usually extracted and summarized. Fortunately, in this study, the adjusted effect size was reported in most of the primary papers where the effect of various confounding variables was controlled. Besides, the possibility of publication bias is another concern in these kinds of meta-analysis studies. Our analyses, however, showed that there is no evidence in favor of publication biases in individual studies. In this study, there was considerable heterogeneity between the included studies. Heterogeneity refers to any variation across primary included articles which mainly include clinical or methodological heterogeneity. No two studies can be identical, so systematic reviews require methods to evaluate the variability of studies to make reasonable decisions about data summarizing and comparisons [30]. Due to significant heterogeneity across the studies, we performed a sub-group analysis (based on study design) and also a random and fixed-effects model to pool the data.

One of the strengths of this study is that unlike a previous meta-analysis that only searched PubMed database a decade ago, three international databases including Scopus, Medline/PubMed, and Web of Science were searched and a general conclusion was made about the association between tubal ligation and breast cancer risk. One of the limitations of this study, however, is that only articles in the English language were reviewed. Moreover, due to the review nature of the study, it was not possible to assess the effect of time (number of years elapsed) since tubal ligation on the risk of breast cancer.

It should be highlighted that combining different study designs and different effect sizes leads to increased heterogeneity between studies, and in some cases, it is not recommended to combine them. In this study, the results of case control and cohort studies were reviewed, and we analyzed the case control (odds ratio) and cohort studies (risk ratio) separately, and because the results of these two types of studies did not differ, the results of these studies were merged as an additional analysis with random-effects model.

In this study, the statistical heterogeneity quantified by the *I*^2^ statistic, and because of a significant heterogeneity, random-effects model preferred to the fixed-effects model. Accordingly, because the number of studies is usually small, there is also the possibility of small study bias that the results should be used with caution.

In this study, some of reported effect size were adjusted for potential confounders and some of them were not adjusted. According to the Cochrane Handbook, the use of adjusted model is preferable if both unadjusted and adjusted intervention effects are reported.

In summary, in this study, we pooled the results of eleven individual studies and our findings did not suggest tubal ligation as a risk factor associated with breast cancer.

## Supplementary Information

**Additional file 1.** Search strategy.

## Data Availability

All data generated or analyzed during this study are included in this published article (and its supplementary information files).

## Abbreviations

SOR: Summary odds ratio
OR: Odds ratio
CI: Confidence interval
SAOR: Summary adjusted odds ratio
IUD: Intrauterine device
MeSH: Medical Subject Headings
PRISMA: Preferred Reporting Items for Systematic Review and Meta-Analysis
