# Supplementary material for: A systematic review and meta-analysis on tubal ligation and breast cancer risk
Source: Syst Rev. 2022 Jun 19;11:126. doi: 10.1186/s13643-022-02000-8 (PMC9208107; doi:10.1186/s13643-022-02000-8)
Supplement: Supplementary file 1 — Additional file 1. Search strategy. [file 13643_2022_2000_MOESM1_ESM.docx]

**Search Date:** November 23, 2020

**PubMed Search**

| Search | Query | Items found |
| --- | --- | --- |
| **#1** | "sterilization, tubal"[MeSH Terms] OR ("sterilization"[All Fields] AND "tubal"[All Fields]) OR "tubal sterilization"[All Fields] OR ("tubal"[All Fields] AND "ligation"[All Fields]) OR "tubal ligation"[All Fields] | [6,203](https://pubmed.ncbi.nlm.nih.gov/?term=Tubal+Ligation&size=20&sort=relevance) |
| **#2** | "sterilization, tubal"[MeSH Terms] OR ("sterilization"[All Fields] AND "tubal"[All Fields]) OR "tubal sterilization"[All Fields] OR ("tubal"[All Fields] AND "occlusion"[All Fields]) OR "tubal occlusion"[All Fields] | [6,028](https://pubmed.ncbi.nlm.nih.gov/?term=Tubal+Occlusion&size=20&sort=relevance) |
| **#3** | "tubal sterilisation"[All Fields] OR "sterilization, tubal"[MeSH Terms] OR ("sterilization"[All Fields] AND "tubal"[All Fields]) OR "tubal sterilization"[All Fields] OR ("tubal"[All Fields] AND "sterilization"[All Fields]) | [5,373](https://pubmed.ncbi.nlm.nih.gov/?term=Tubal+Sterilization&size=20&sort=relevance) |
| **#4** | "female sterilisation"[All Fields] OR "sterilization, reproductive"[MeSH Terms] OR ("sterilization"[All Fields] AND "reproductive"[All Fields]) OR "reproductive sterilization"[All Fields] OR ("female"[All Fields] AND "sterilization"[All Fields]) OR "female sterilization"[All Fields] | [18,595](https://pubmed.ncbi.nlm.nih.gov/?term=Female+Sterilization&size=20&sort=relevance) |
| **#5 (#1 OR #2 OR #3 OR #4)** | "female sterilisation"[All Fields] OR "sterilization, reproductive"[MeSH Terms] OR ("sterilization"[All Fields] AND "reproductive"[All Fields]) OR "reproductive sterilization"[All Fields] OR ("female"[All Fields] AND "sterilization"[All Fields]) OR "female sterilization"[All Fields] OR ("tubal sterilisation"[All Fields] OR "sterilization, tubal"[MeSH Terms] OR ("sterilization"[All Fields] AND "tubal"[All Fields]) OR "tubal sterilization"[All Fields] OR ("tubal"[All Fields] AND "sterilization"[All Fields])) OR ("sterilization, tubal"[MeSH Terms] OR ("sterilization"[All Fields] AND "tubal"[All Fields]) OR "tubal sterilization"[All Fields] OR ("tubal"[All Fields] AND "occlusion"[All Fields]) OR "tubal occlusion"[All Fields]) OR ("sterilization, tubal"[MeSH Terms] OR ("sterilization"[All Fields] AND "tubal"[All Fields]) OR "tubal sterilization"[All Fields] OR ("tubal"[All Fields] AND "ligation"[All Fields]) OR "tubal ligation"[All Fields]) | [20,167](https://pubmed.ncbi.nlm.nih.gov/?term=%28%28%28Female+Sterilization%29+OR+%28Tubal+Sterilization%29%29+OR+%28Tubal+Occlusion%29%29+OR+%28Tubal+Ligation%29&sort=relevance&size=20) |
| **#6** | "breast neoplasms"[MeSH Terms] OR ("breast"[All Fields] AND "neoplasms"[All Fields]) OR "breast neoplasms"[All Fields] OR ("breast"[All Fields] AND "cancer"[All Fields]) OR "breast cancer"[All Fields] | [422,252](https://pubmed.ncbi.nlm.nih.gov/?term=Breast+Cancer&size=20&sort=relevance) |
| **#7 (#5 AND #6)** | ("breast neoplasms"[MeSH Terms] OR ("breast"[All Fields] AND "neoplasms"[All Fields]) OR "breast neoplasms"[All Fields] OR ("breast"[All Fields] AND "cancer"[All Fields]) OR "breast cancer"[All Fields]) AND ("sterilization, tubal"[MeSH Terms] OR ("sterilization"[All Fields] AND "tubal"[All Fields]) OR "tubal sterilization"[All Fields] OR ("tubal"[All Fields] AND "ligation"[All Fields]) OR "tubal ligation"[All Fields] OR ("tubal"[All Fields] AND "occlusion"[All Fields]) OR "tubal occlusion"[All Fields] OR "tubal sterilisation"[All Fields] OR ("tubal"[All Fields] AND "sterilization"[All Fields]) OR "female sterilisation"[All Fields] OR "sterilization, reproductive"[MeSH Terms] OR ("sterilization"[All Fields] AND "reproductive"[All Fields]) OR "reproductive sterilization"[All Fields] OR ("female"[All Fields] AND "sterilization"[All Fields])) | [202](https://pubmed.ncbi.nlm.nih.gov/?term=%28Breast+Cancer%29+AND+%28%22sterilization%2C+tubal%22%5BMeSH+Terms%5D+OR+%28%22sterilization%22%5BAll+Fields%5D+AND+%22tubal%22%5BAll+Fields%5D%29+OR+%22tubal+sterilization%22%5BAll+Fields%5D+OR+%28%22tubal%22%5BAll+Fields%5D+AND+%22ligation%22%5BAll+Fields%5D%29+OR+%22tubal+ligation%22%5BAll+Fields%5D+OR+%28%22tubal%22%5BAll+Fields%5D+AND+%22occlusion%22%5BAll+Fields%5D%29+OR+%22tubal+occlusion%22%5BAll+Fields%5D+OR+%22tubal+sterilisation%22%5BAll+Fields%5D+OR+%28%22tubal%22%5BAll+Fields%5D+AND+%22sterilization%22%5BAll+Fields%5D%29+OR+%22female+sterilisation%22%5BAll+Fields%5D+OR+%22sterilization%2C+reproductive%22%5BMeSH+Terms%5D+OR+%28%22sterilization%22%5BAll+Fields%5D+AND+%22reproductive%22%5BAll+Fields%5D%29+OR+%22reproductive+sterilization%22%5BAll+Fields%5D+OR+%28%22female%22%5BAll+Fields%5D+AND+%22sterilization%22%5BAll+Fields%5D%29%29&sort=relevance&size=20) |
| #8 (#7 Filters: **Humans, English)** | ("breast neoplasms"[MeSH Terms] OR ("breast"[All Fields] AND "neoplasms"[All Fields]) OR "breast neoplasms"[All Fields] OR ("breast"[All Fields] AND "cancer"[All Fields]) OR "breast cancer"[All Fields]) AND ("sterilization, tubal"[MeSH Terms] OR ("sterilization"[All Fields] AND "tubal"[All Fields]) OR "tubal sterilization"[All Fields] OR ("tubal"[All Fields] AND "ligation"[All Fields]) OR "tubal ligation"[All Fields] OR ("tubal"[All Fields] AND "occlusion"[All Fields]) OR "tubal occlusion"[All Fields] OR "tubal sterilisation"[All Fields] OR ("tubal"[All Fields] AND "sterilization"[All Fields]) OR "female sterilisation"[All Fields] OR "sterilization, reproductive"[MeSH Terms] OR ("sterilization"[All Fields] AND "reproductive"[All Fields]) OR "reproductive sterilization"[All Fields] OR ("female"[All Fields] AND "sterilization"[All Fields])) | [160](https://pubmed.ncbi.nlm.nih.gov/?term=%28Breast+Cancer%29+AND+%28%22sterilization%2C+tubal%22%5BMeSH+Terms%5D+OR+%28%22sterilization%22%5BAll+Fields%5D+AND+%22tubal%22%5BAll+Fields%5D%29+OR+%22tubal+sterilization%22%5BAll+Fields%5D+OR+%28%22tubal%22%5BAll+Fields%5D+AND+%22ligation%22%5BAll+Fields%5D%29+OR+%22tubal+ligation%22%5BAll+Fields%5D+OR+%28%22tubal%22%5BAll+Fields%5D+AND+%22occlusion%22%5BAll+Fields%5D%29+OR+%22tubal+occlusion%22%5BAll+Fields%5D+OR+%22tubal+sterilisation%22%5BAll+Fields%5D+OR+%28%22tubal%22%5BAll+Fields%5D+AND+%22sterilization%22%5BAll+Fields%5D%29+OR+%22female+sterilisation%22%5BAll+Fields%5D+OR+%22sterilization%2C+reproductive%22%5BMeSH+Terms%5D+OR+%28%22sterilization%22%5BAll+Fields%5D+AND+%22reproductive%22%5BAll+Fields%5D%29+OR+%22reproductive+sterilization%22%5BAll+Fields%5D+OR+%28%22female%22%5BAll+Fields%5D+AND+%22sterilization%22%5BAll+Fields%5D%29%29&filter=hum_ani.humans&filter=lang.english&size=20&sort=relevance) |

**Scopus**

Bottom of Form

| History Count | Search Terms | Results |
| --- | --- | --- |
| 4 | ( ( TITLE-ABS-KEY ( "Tubal ligation" )  OR  TITLE-ABS-KEY ( "Tubal Sterilization" )  OR  TITLE-ABS-KEY ( "Tubal Sterilisation" )  OR  TITLE-ABS-KEY ( "tubal occlusion" )  OR  TITLE-ABS-KEY ( "female sterilization" )  OR  TITLE-ABS-KEY ( "female sterilisation" )  OR  TITLE-ABS-KEY ( "reproductive sterilization" )  OR  TITLE-ABS-KEY ( "reproductive sterilisation" ) ) )  AND  ( ( TITLE-ABS-KEY ( "Breast cancer" )  OR  TITLE-ABS-KEY ( "breast neoplasms" )  OR  TITLE-ABS-KEY ( breast  AND cancer ) ) )  AND  ( LIMIT-TO ( LANGUAGE ,  "English" ) ) | [165 document results](https://www.scopus.com/search/history/results.uri?origin=searchhistory&shid=5) |
| 3 | ( ( TITLE-ABS-KEY ( "Tubal ligation" )  OR  TITLE-ABS-KEY ( "Tubal Sterilization" )  OR  TITLE-ABS-KEY ( "Tubal Sterilisation" )  OR  TITLE-ABS-KEY ( "tubal occlusion" )  OR  TITLE-ABS-KEY ( "female sterilization" )  OR  TITLE-ABS-KEY ( "female sterilisation" )  OR  TITLE-ABS-KEY ( "reproductive sterilization" )  OR  TITLE-ABS-KEY ( "reproductive sterilisation" ) ) )  AND  ( ( TITLE-ABS-KEY ( "Breast cancer" )  OR  TITLE-ABS-KEY ( "breast neoplasms" )  OR  TITLE-ABS-KEY ( breast  AND cancer ) ) ) | [177 document results](https://www.scopus.com/search/history/results.uri?origin=searchhistory&shid=4) |
| 2 | (TITLE-ABS-KEY ( "Breast cancer" )  OR  TITLE-ABS-KEY ( "breast neoplasms" )  OR  TITLE-ABS-KEY ( breast  AND cancer ) ) | [559,237 document results](https://www.scopus.com/search/history/results.uri?origin=searchhistory&shid=3) |
| 1 | ( TITLE-ABS-KEY ( "Tubal ligation" )  OR  TITLE-ABS-KEY ( "Tubal Sterilization" )  OR  TITLE-ABS-KEY ( "Tubal Sterilisation" )  OR  TITLE-ABS-KEY ( "tubal occlusion" )  OR  TITLE-ABS-KEY ( "female sterilization" )  OR  TITLE-ABS-KEY ( "female sterilisation" )  OR  TITLE-ABS-KEY ( "reproductive sterilization" )  OR  TITLE-ABS-KEY ( "reproductive sterilisation" ) ) | [8,924 document results](https://www.scopus.com/search/history/results.uri?origin=searchhistory&shid=2) |

**ISI**

| # 3 | [139](https://apps.webofknowledge.com/summary.do?product=WOS&doc=1&qid=17&SID=C3rFlJGlssKmPFnZB2H&search_mode=CombineSearches&update_back2search_link_param=yes) | #2  AND  #1  *Indexes=SCI-EXPANDED, SSCI, A&HCI, CPCI-S, CPCI-SSH, ESCI Timespan=All years* |
| --- | --- | --- |
| # 2 | [**568,979**](https://apps.webofknowledge.com/summary.do?product=WOS&doc=1&qid=16&SID=C3rFlJGlssKmPFnZB2H&search_mode=GeneralSearch&update_back2search_link_param=yes) | **TOPIC:**  ("Breast cancer")  *OR*  **TOPIC:**  ("Breast neoplasms")  *OR*  **TOPIC:**  (Breast cancer)  *Indexes=SCI-EXPANDED, SSCI, A&HCI, CPCI-S, CPCI-SSH, ESCI Timespan=All years* |
| # 1 | [**3,892**](https://apps.webofknowledge.com/summary.do?product=WOS&doc=1&qid=15&SID=C3rFlJGlssKmPFnZB2H&search_mode=GeneralSearch&update_back2search_link_param=yes) | **TOPIC:**  ("Tubal ligation")  *OR*  **TOPIC:**  ("Tubal Sterilization")  *OR*  **TOPIC:**  ("Tubal Sterilisation")  *OR*  **TOPIC:**  ("tubal occlusion")  *OR*  **TOPIC:**  ("female sterilization")  *OR*  **TOPIC:**  ("female sterilisation")  *OR*  **TOPIC:**  ("reproductive sterilization")  *OR*  **TOPIC:**  ("reproductive sterilisation")  *Indexes=SCI-EXPANDED, SSCI, A&HCI, CPCI-S, CPCI-SSH, ESCI Timespan=All years* |
